# Long-Term Outcome of Patients with a Floating Hip Injury of Müller Type A: An Analysis of Prognostic Factors Linked to Functional Outcomes

**DOI:** 10.3390/jcm15093321

**Published:** 2026-04-27

**Authors:** Beytullah Unat, Cagrı Karabulut, Musa Alperen Bilgin, Ramazan Erol, Ilkan Kisi, Ibrahim Halil Rızvanoglu, Nevzat Gönder

**Affiliations:** 1 Department of Orthopaedics and Traumatology, School of Medicine, Gaziantep Islam Science and Technology University, Gaziantep 27000, Turkey; unatbeytullah@gmail.com; 2 Department of Orthopaedics and Traumatology, T.C. Ministry of Health Pazarcık State Hospital, Kahramanmaraş 46700, Turkey; 3 Department of Orthopaedics and Traumatology, T.C. Ministry of Health Aksaray Training and Research Hospital, Aksaray 68000, Turkey; alpperren@gmail.com; 4 Department of Orthopaedics and Traumatology, Faculty of Medicine, Gaziantep University, Gaziantep 27310, Turkey; ramazanerol0808@gmail.com (R.E.); ilkan-kisi1234@hotmail.com (I.K.); 5 Department of Orthopaedics and Traumatology, Private NCR International Hospital, Gaziantep 27000, Turkey; dr.rizvanoglu@gmail.com

**Keywords:** floating hip, acetabular fracture, femoral fracture, Matta’s radiological score, Majeed score, prognostic factors, polytrauma

## Abstract

**Background/Objectives**: A floating hip injury, defined as an ipsilateral fracture of the pelvis or acetabulum combined with a femoral fracture, represents a rare and devastating musculoskeletal injury resulting from high-energy trauma. Although Müller type A floating hip injuries comprising an acetabular fracture with an ipsilateral femoral fracture are recognized for their clinical complexity, the long-term prognostic factors influencing functional outcomes remain poorly elucidated. This study aimed to identify independent prognostic factors associated with unsatisfactory long-term functional outcomes in patients with Müller type A floating hip injuries. **Methods**: A retrospective study was performed on 68 consecutive patients with Müller type A floating hip injuries who underwent surgical fixation at a single tertiary trauma center, with a minimum follow-up period of 5 years. Functional outcomes were assessed using the Majeed score, and patients were dichotomized into satisfactory (n = 48; 70.6%) and unsatisfactory (n = 20; 29.4%) outcome groups. Acetabular fractures were classified according to the Judet–Letournel system, and femoral fractures were classified by fracture level (proximal, shaft, or distal). Radiological outcomes were evaluated using Matta’s radiological grading system. Demographic, injury-specific, and treatment-related variables were compared between groups using the Mann–Whitney U test and chi-square test with Bonferroni correction. A multivariate binary logistic regression model was constructed to determine independent predictors of unsatisfactory outcomes. **Results**: The mean age was 37.15 ± 12.07 years, with a male predominance (67.6%). The predominant mechanism of injury was pedestrian struck by vehicle (54.4%), followed by motor vehicle collision (27.9%) and fall from height (17.6%); collectively, high-energy vehicular trauma accounted for 82.3% of cases. In the univariate analysis, transverse with posterior wall acetabular fracture pattern (*p* = 0.001), proximal femur fracture level (*p* = 0.001), associated lower extremity fractures (*p* = 0.001), nerve damage (*p* = 0.001), higher body mass index (BMI) (*p* = 0.001), and lower Matta’s radiological scores (*p* = 0.001) were significantly associated with unsatisfactory outcomes. Three independent predictors emerged in the multivariate logistic regression: BMI (OR = 1.50; 95% CI: 1.05–2.15; *p* = 0.025), the presence of associated lower extremity fractures (OR = 29.02; 95% CI: 2.83–297.67; *p* = 0.005), and Matta’s radiological score (OR = 0.06; 95% CI: 0.01–0.56; *p* = 0.014). The model yielded internal discriminatory metrics within the acceptable range (overall accuracy 89.7%, sensitivity 95.8%, specificity 75.0%, Nagelkerke R^2^ = 0.757); however, given the limited events-per-variable ratio (~6.7) and the wide confidence intervals observed for some predictors, these internal performance estimates are likely optimistic due to potential overfitting, and the findings should be interpreted as exploratory pending external validation. **Conclusions**: Elevated BMI, the presence of associated ipsilateral lower extremity fractures, and poor quality of acetabular reduction, assessed via Matta’s radiological criteria, are independent determinants of unsatisfactory long-term functional outcomes in Müller type A floating hip injuries. These findings underscore the critical importance of achieving anatomical reduction in the acetabulum and highlight the compounding effect of additional ipsilateral limb injuries on patient prognosis.

## 1. Introduction

A floating hip injury constitutes an ipsilateral fracture of the pelvic ring or acetabulum combined with a femoral fracture, resulting in skeletal discontinuity proximal and distal to the hip joint. First described by Liebergall et al. in 1992, this injury pattern was subsequently refined by Müller et al. in 1999 into three subtypes: type A, involving an acetabular fracture with an ipsilateral femoral fracture; type B, involving a pelvic ring fracture with an ipsilateral femoral fracture; and type C, encompassing combined pelvic ring and acetabular fractures with an ipsilateral femoral fracture [1,2]. These injuries are characteristically the result of high-energy trauma, including motor vehicle collisions and falls from significant height, and are frequently observed in young males [3,4].

The estimated incidence of floating hip injuries is approximately 1 per 10,000 fractures, occurring only 2 to 8 times annually even in high-volume trauma centers [2,5,6]. Concomitant injuries to the chest, abdomen, brain, and other extremities are common, and injury severity scores (ISSs) consistently exceed 20 in reported series [3,7,8]. Yang et al., in their comprehensive review of the literature, emphasized that the combination of pelvic or acetabular and femoral fractures produces a multiplicative rather than purely additive injury burden, compounding the risk of hemodynamic instability, prolonged hospital stays, and adverse functional outcomes [9].

The principles of damage control orthopedics (DCO) govern the acute management of these polytraumatized patients, with femoral fixation generally preceding acetabular reconstruction [4,10]. The few available studies have primarily focused on type B floating hip injuries or have combined all subtypes without distinction, thereby obscuring the unique prognostic landscape of each injury pattern [6,11]. Kokubo et al. identified nerve damage, associated lower extremity fractures, and conservative treatment as determinants of short-term functional outcome in unstable pelvic ring fractures, while nerve damage and residual radiological displacement were the primary determinants of long-term outcome [12]. Similarly, Wong et al. demonstrated that floating hip cases had significantly higher rates of postoperative complications (53.8% vs. 20.0%; *p* = 0.025) and longer hospital stays, when compared with matched controls with isolated pelvic or acetabular fractures [13].

The quality of acetabular reduction, assessed via Matta’s radiological criteria, has long been recognized as a pivotal determinant of the outcome following isolated acetabular fracture surgery [14,15]. Meena et al. reported that clinical and radiological outcomes at the latest follow-up were excellent or good in more than 60% of type B floating hip cases, with a significant association between reduction quality on postoperative radiographs and functional outcomes [11]. Gänsslen et al. demonstrated that, even in anatomically reconstructed acetabular joints, transverse with posterior wall fractures carry a significant risk of early joint failure, with a 32.8% joint failure rate [16]. However, the specific contribution of radiological reduction quality to long-term outcomes in the context of floating hip injuries has not been systematically evaluated.

The role of patient-related factors in determining outcomes following complex periarticular injuries has garnered increasing attention. Obesity is recognized as an independent risk factor for surgical complications and poorer functional outcomes in orthopedic trauma, yet its specific influence on the prognosis of floating hip injuries remains largely unexplored [17]. Among the three Müller subtypes, type A injuries, defined by the combination of an acetabular fracture with an ipsilateral femoral fracture, represent a particularly challenging surgical entity; nevertheless, no prior study has simultaneously and systematically examined the combined contributions of patient anthropometric factors, injury complexity, and radiological reduction quality as independent prognostic determinants specifically within this subtype. This gap limits evidence-based patient counseling, surgical decision making, and prognostic risk stratification in this population.

This study aimed to identify independent prognostic factors associated with unsatisfactory long-term functional outcomes in patients with Müller type A floating hip injuries.

## 2. Materials and Methods

### 2.1. Study Design and Patient Selection

A retrospective cohort analysis was conducted at a single tertiary trauma center following institutional review board approval. The inclusion criteria required surgical fixation for a Müller type A floating hip injury between January 2000 and December 2019, with a minimum follow-up of five years. The exclusion criteria included patients who died within 48 h of injury, those whose pelvic or acetabular injuries were managed conservatively, patients with Pipkin type IV fractures (femoral head fractures with concomitant acetabular fractures), isolated iliac wing fractures not affecting pelvic ring integrity, open fractures, and patients with incomplete clinical or radiological data. A total of 68 patients met the inclusion criteria and formed the study cohort.

This study was performed in line with the principles of the Declaration of Helsinki. Approval was granted by the Gaziantep University Non-Interventional Research Ethics Committee (date: 17 December 2025/No. 2025/457). Informed consent was obtained from all participants involved in this study.

### 2.2. Clinical Assessment and Data Collection

Patient demographics, injury characteristics, and treatment-related variables were retrospectively extracted from institutional electronic medical records, operative notes, and postoperative imaging archives. Data completeness was verified by cross-referencing with the hospital administrative database; patients with missing key clinical or radiological variables were excluded from the study cohort. The demographic variables included age, sex, and BMI. Injury-specific variables included mechanism of injury (motor vehicle collision, pedestrian struck by vehicle, and fall from height), side of injury, acetabular fracture type (classified according to the Judet–Letournel system), femoral fracture level (classified as proximal, shaft, or distal), associated ipsilateral lower extremity fractures, associated upper extremity fractures, nerve damage, vascular damage, and concomitant injuries to the brain, spine, thorax, abdomen, and genitourinary system. Ipsilateral and contralateral knee ligament injuries, smoking status, time to definitive surgery, and length of hospital stay were also recorded.

Injury severity was assessed using the revised trauma score (RTS) (Table 1). Comorbidity was assessed using the age-adjusted Charlson comorbidity index (ACCI). The quality of acetabular reduction was evaluated on postoperative radiographs using Matta’s radiological scoring system, which was scored as anatomical (0–1 mm residual displacement), imperfect reduction (2–3 mm), and poor reduction (>3 mm) [14]. For regression analysis, Matta’s score was dichotomized as satisfactory (anatomical or imperfect) versus unsatisfactory (poor). Functional outcomes were assessed at final follow-up using the Majeed score, a validated instrument encompassing the domains of pain, work performance, sitting tolerance, sexual function, and walking ability [18]. Patients were dichotomized into satisfactory (Majeed score ≥ 70; “good” group; n = 48) and unsatisfactory (Majeed score < 70; “poor” group; n = 20) outcome groups for comparative analysis [18]. This threshold is consistent with Majeed’s original scoring classification, in which a score of 70–80 corresponds to a “good” outcome, and has been employed as the dichotomization cutoff in multiple prior studies of pelvic and acetabular fracture outcomes [18]. The Majeed score was analyzed as a continuous variable in the descriptive analysis. Dichotomization was applied solely for logistic regression to enable identification of independent predictors of clinically meaningful outcome groups.

Complications were classified as femoral head avascular necrosis (AVN), heterotopic ossification, nonunion, post-traumatic coxarthrosis, post-traumatic gonarthrosis, wound infection, and osteomyelitis.

### 2.3. Statistical Analysis

The normality of distribution of continuous variables was assessed using the Shapiro–Wilk test. As the data were not normally distributed, the Mann–Whitney U test was employed for between-group comparisons of continuous variables. The chi-square test was used to evaluate associations between categorical variables, with Bonferroni correction applied for multiple comparisons. Variables that demonstrated statistical significance in univariate analysis (*p* < 0.05) were assessed for collinearity using variance inflation factor (VIF) analysis (with VIF > 2.5 indicating unacceptable collinearity). Variables meeting the prerequisite assumptions were entered into a multivariate binary logistic regression model to identify independent predictors of unsatisfactory functional outcome.

The Majeed score was excluded from the regression model on conceptual grounds, as it constitutes the dependent variable used to define the binary outcome (satisfactory vs. unsatisfactory); its inclusion as a predictor would represent circular reasoning. Accordingly, the Majeed score also demonstrated collinearity with Matta’s radiological score during preliminary variable screening (VIF = 2.592), which is reported here for methodological transparency. Model performance was evaluated using the Cox and Snell R^2^, Nagelkerke R^2^, sensitivity, specificity, and overall classification accuracy. All statistical analyses were performed using SPSS for Windows version 22.0 (IBM Corp., Armonk, NY, USA), and a two-tailed *p*-value of < 0.05 was considered statistically significant.

Given the rarity of Müller type A floating hip injuries (with an estimated incidence of approximately one per 10,000 fractures, occurring only two to eight times annually even in high-volume trauma centers), a formal a priori power calculation was not feasible. The cohort of 68 patients represents the complete series of eligible patients treated at our institution over 20 years and constitutes, to our knowledge, one of the largest single-center series reported specifically for this injury subtype. The events-per-variable (EPV) ratio in the multivariate logistic regression model was approximately 6.7 (20 outcome events across 3 independent predictors), which is below the conventionally recommended threshold of 10. This limitation is explicitly acknowledged and reflected in the wide confidence intervals observed for certain predictors.

## 3. Results

### 3.1. Patient Demographics and Injury Characteristics

The cohort comprised 68 patients (46 males and 22 females), with a mean age of 37.15 ± 12.07 years (range: 20–64 years) and a mean BMI of 25.46 ± 3.76 kg/m^2^ (range: 20.0–35.6 kg/m^2^). The mean revised trauma score was 6.87 ± 0.83, and the mean age-adjusted Charlson comorbidity index was 2.07 ± 1.55. The right side was involved in 40 (58.8%) patients and the left side in 28 (41.2%). The predominant mechanism of injury was pedestrian struck by vehicle (54.4%), followed by motor vehicle collision (27.9%) and fall from height (17.6%).

**Figure 1:**
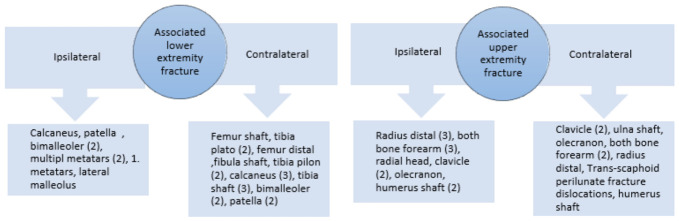
Distribution of associated lower and upper extremity fractures in the study cohort (n = 68).

**Figure 2:**
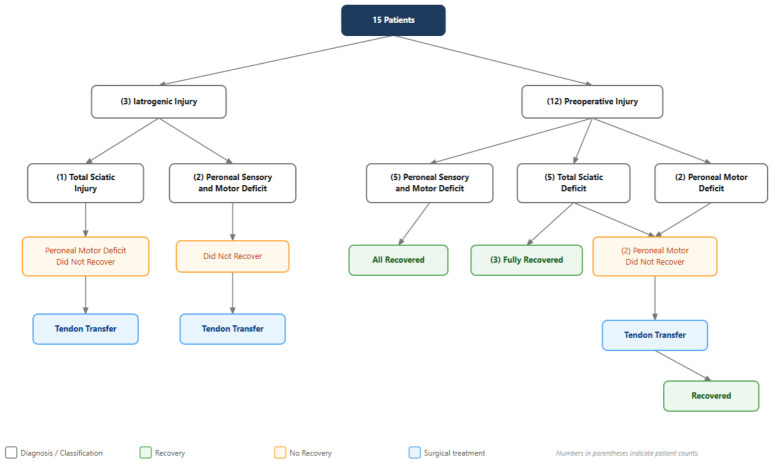
Clinical course and treatment algorithm for 15 patients with sciatic and/or peroneal nerve injury: the management strategy employed and the rate of neurological recovery at final follow-up.

The most common acetabular fracture pattern was transverse with posterior wall (27.9%), followed by anterior column (14.7%), both columns (13.2%), and posterior wall (13.2%) (Table 2). Fracture–dislocations were identified in five patients, all of whom underwent emergent closed reduction in the emergency department upon presentation. Femoral fractures were located at the shaft level in 32 patients (47.1%), the proximal level in 26 (38.2%), and the distal level in 10 (14.7%). Associated ipsilateral lower extremity fractures were present in 25 patients (36.8%) and associated upper extremity fractures in 21 (30.9%) (Figure 1). As illustrated in Figure 1, lower extremity fractures predominantly involved the tibia, calcaneus, talus, and foot, while upper extremity fractures included radial, humeral, and hand injuries. The distribution highlights the polytraumatic burden carried by this patient population, with a substantial proportion sustaining injuries to multiple limb segments simultaneously. Nerve injuries were present in 15 patients (22.1%) (Figure 2). As depicted in Figure 2, nerve injuries involved the sciatic nerve in most cases, with a subset affecting the peroneal division exclusively. Management followed a standardized algorithm; in particular, patients with incomplete nerve lesions were observed conservatively with serial neurological examination, while those with complete lesions unresponsive to conservative management at one year underwent tendon transfer. Vascular injuries were present in three patients (4.4%), and iatrogenic injuries were sustained by two patients to the iliac vein and by one patient to the femoral vein; however, these were successfully repaired and managed without complications.

The number of fractures is indicated by the numbers in brackets. Lower extremity fractures included tibial, patellar, and foot injuries; upper extremity fractures included humeral, forearm, and hand injuries. Patients with multiple concomitant fractures were counted once per anatomical region.

Concomitant thoracic injuries were documented in 25 patients (36.8%), abdominal injuries in 16 (23.5%), brain injuries in 15 (22.1%), spinal injuries in 11 (16.2%), and genitourinary injuries in 11 (16.2%). Ipsilateral knee ligament injuries were observed in 14 patients (20.6%) (*p* = 0.561) and contralateral knee ligament injuries in 3 (4.4%) (*p* = 0.305). Eighteen patients (26.5%) were active smokers (Table 3). In our study, only five (7.3%) individuals presented with isolated floating hip injuries; all other patients had concurrent organ or system injuries.

### 3.2. Surgical Procedure

A staged surgical approach was employed for the management of these injuries. In hemodynamically stable patients, femoral fixation was performed upon admission as the initial procedure, followed by acetabular fixation in a subsequent operative session. In patients presenting with hemodynamic instability, temporary stabilization was achieved using external fixation for the femoral shaft and distal femoral fractures or skeletal traction via tibial pin for proximal femoral fractures. Definitive surgical intervention was deferred until physiological parameters were optimized. Once hemodynamic stability was restored, both fractures were addressed in a single operative session. In cases requiring simultaneous fixation, the surgical sequence was determined according to the status of the femoral head; in particular, acetabular fixation was prioritized when the femoral head was dislocated and could not be concentrically reduced whereas, in all other cases, femoral fixation preceded acetabular reconstruction. Acetabular fractures were stabilized using 3.5 mm reconstruction plates, supplemented with one-third tubular plates for the fixation of small fragments when deemed necessary. Femoral fractures were managed with fixation methods selected according to the fracture level: intramedullary nailing for diaphyseal fractures, a dynamic hip screw (DHS) or proximal femoral nail (PFN) for intertrochanteric fractures, plate osteosynthesis for distal fractures, and cannulated screw fixation for femoral neck fractures (Table 4). Postoperative rehabilitation followed a standardized institutional protocol. All patients were mobilized non-weight bearing for the first six weeks following definitive femoral fixation. Partial weight bearing was initiated at six weeks, and progression to full weight bearing was permitted at three months, subject to satisfactory clinical and radiological assessment at each follow-up visit. Patients with associated ipsilateral lower extremity fractures underwent rehabilitation planning coordinated with the treating physiotherapy team, with weight-bearing timelines adjusted according to the fixation stability of all injured segments.

### 3.3. Functional and Radiological Outcomes

At the final follow-up, the mean Majeed Score was 73.88 ± 10.71 (range: 54–88). Forty-eight patients (70.6%) achieved satisfactory functional outcomes (mean Majeed score: 79.63 ± 6.54), while 20 patients (29.4%) had unsatisfactory outcomes (mean Majeed score: 60.10 ± 3.89; *p* = 0.001). BMI was significantly higher in the unsatisfactory group (29.09 ± 2.84 vs. 23.95 ± 2.98 kg/m^2^; *p* = 0.001). According to Matta’s radiological criteria, the satisfactory group reported 42 cases of anatomical reduction, 2 cases of imperfect reduction, and 4 cases of poor reduction. Matta’s radiological score was significantly higher in the satisfactory group (*p* = 0.001). The unsatisfactory group comprised five cases of anatomical reduction, nine cases of imperfect reduction, and six cases of poor reduction. No significant between-group differences were observed for age (*p* = 0.762), revised trauma score (*p* = 0.506), age-adjusted Charlson comorbidity index (*p* = 0.506), time to definitive surgery (*p* = 0.460), or length of hospital stay (*p* = 0.568) (Table 5).

Regarding complications, two patients suffered avascular necrosis (AVN) of the femoral head, four had nonunion, four had gonarthrosis, three suffered from coxarthrosis, two had osteomyelitis, six encountered surgical site infections, two had a Morel–Lavallée lesion on the thigh, and seven were diagnosed with heterotopic ossification (HO). No statistically significant difference in complications was seen between the groups (*p* = 0.135). Three individuals suffered deep vein thrombosis, although they recovered without problems. None of the patients had pulmonary thromboembolism. HO was observed in seven patients, of whom three were classified as Brooker Type 2 and four as Brooker Type 1. All cases of HO demonstrated spontaneous regression without requiring surgical intervention during the follow-up period. Femoral nonunion occurred in four patients: three involved the femoral shaft, and one involved the distal femur. Shaft nonunions were managed with exchange intramedullary nailing, whereas the distal femoral nonunion was treated with implant removal followed by repeat osteosynthesis with plate fixation and autologous iliac crest bone grafting. Osseous union was ultimately achieved in all cases.

Among the two patients who developed AVN of the femoral head, one progressed to Ficat–Arlet Type 3 at the most recent follow-up and continued to experience unsatisfactory clinical outcomes attributable to persistent hip and thigh pain. The remaining patient with AVN underwent total hip arthroplasty (THA). Gonarthrosis developed in four patients who had undergone surgical management of distal femoral fractures; three were classified as Kellgren–Lawrence Grade 2 and one as Grade 4. The patient with Grade 4 gonarthrosis was subsequently treated with total knee arthroplasty (TKA). Coxarthrosis was identified in three patients, all of whom underwent THA.

Regarding infectious complications, four patients presented with superficial wound infections that resolved with systemic antibiotic therapy and serial wound dressings. Deep wound infection occurred in two patients: one following surgical management of a transverse with posterior wall acetabular fracture, localized to the posterior incision site, and the other following open reduction in an intertrochanteric femoral fracture. Both deep infections were successfully managed with serial surgical debridement and targeted antibiotic therapy. Two patients who developed Morel–Lavallée lesions were treated with percutaneous drainage and antibiotic therapy, with complete resolution. Osteomyelitis developed in two patients who had undergone surgical fixation of femoral shaft fractures. In both cases, the implants were removed following confirmation of osseous union, and the patients were managed with serial surgical debridement and antibiotic therapy, achieving full recovery without further complications.

### 3.4. Univariate Analysis of Categorical Variables

Acetabular fracture type was significantly associated with outcome (*p* = 0.001) in the univariate analysis of categorical variables. Bonferroni-corrected post hoc analysis revealed that the posterior wall fracture pattern was significantly more prevalent in the satisfactory group (18.8% vs. 0%; *p* = 0.049), whereas the transverse with posterior wall pattern was significantly more prevalent in the unsatisfactory group (65.0% vs. 12.5%; *p* < 0.001). Femoral fracture level was also significantly associated with outcome (*p* = 0.001), with proximal femur fractures predominating in the unsatisfactory group (75.0% vs. 22.9%) and shaft fractures predominating in the satisfactory group (58.3% vs. 20.0%). The presence of associated lower extremity fractures (80.0% vs. 18.8%; *p* = 0.001) and nerve damage (55.0% vs. 8.3%; *p* = 0.001) was significantly more prevalent in the unsatisfactory group. The motor vehicle collision injury mechanism was also more frequent in the unsatisfactory group (50.0% vs. 18.8%; *p* = 0.031). Gender, side, associated upper extremity fractures, vascular damage, brain injury, spinal injury, thoracic injury, abdominal injury, genitourinary injury, ipsilateral and contralateral knee ligament injuries, smoking habit, and complications were not significantly associated with outcome (all *p* > 0.05).

### 3.5. Multivariate Logistic Regression Analysis

Following collinearity assessment (all VIF values < 2.6), the multivariate binary logistic regression model incorporated femoral fracture level, associated lower extremity fracture, nerve damage, fracture mechanism, Matta’s radiological score, and BMI as candidate predictors (Table 6). The Majeed score was excluded from the model on conceptual grounds, as it defines the binary dependent variable (satisfactory vs. unsatisfactory outcome) and its inclusion as a predictor would constitute circular reasoning. Its collinearity with Matta’s radiological score during preliminary screening (VIF = 2.592) is noted for transparency. The final model identified three independent predictors of unsatisfactory functional outcome (Table 7): (1) BMI (OR = 1.50; 95% CI: 1.05–2.15; *p* = 0.025), indicating that each one-unit increase in BMI was associated with a 1.5-fold increase in the odds of an unsatisfactory outcome; (2) the presence of associated lower extremity fractures (OR = 29.02; 95% CI: 2.83–297.67; *p* = 0.005), representing a substantially elevated but imprecisely estimated odds of an unsatisfactory outcome; and (3) Matta’s radiological score (OR = 0.06; 95% CI: 0.01–0.56; *p* = 0.014), indicating that a satisfactory radiological reduction was associated with a marked reduction in the odds of an unsatisfactory outcome (Table 7).

The Majeed score was excluded from the regression model because it constitutes the dependent variable (outcome measure); its collinearity with Matta’s radiological score (VIF = 2.592) during preliminary screening is reported for methodological transparency. Candidate variables entered into the model: femoral fracture level, associated lower extremity fracture, nerve damage, fracture mechanism, Matta’s radiological score, and BMI.

Model performance: Cox and Snell R^2^ = 0.532; Nagelkerke R^2^ = 0.757; sensitivity = 95.8%; specificity = 75.0%; and overall accuracy = 89.7%. Independent predictors that achieved statistical significance in the final model: BMI, associated lower extremity fracture, and Matta’s radiological score. OR: odds ratio; CI: confidence interval.

The model demonstrated acceptable discriminatory performance in the internal dataset, although the limited events-per-variable ratio (~6.7) and the wide confidence intervals observed for some predictors warrant an exploratory interpretation of the effect size estimates. The Cox and Snell R^2^ was 0.532, the Nagelkerke R^2^ was 0.757, the sensitivity was 95.8%, the specificity was 75.0%, and the overall classification accuracy was 89.7%. The Hosmer–Lemeshow goodness-of-fit test was not performed, as this test is known to yield unreliable results in small samples with highly discriminating models generating extreme predicted probabilities. Therefore, discrimination metrics are reported as the primary indicators of model performance.

## 4. Discussion

This study represents one of the largest single-center analyses of prognostic factors determining long-term functional outcomes in Müller type A floating hip injuries. Our multivariate analysis identified three independent predictors of unsatisfactory outcomes: elevated body mass index, the presence of associated ipsilateral lower extremity fractures, and poor quality of acetabular reduction, as measured with Matta’s radiological scoring system. These findings provide clinically actionable prognostic information for a complex injury that remains poorly characterized in the orthopedic literature. In our study, only five (7.3%) individuals presented with isolated floating hip injuries; all other patients had concurrent organ or system injuries. This injury pattern should additionally be considered as a complex polytrauma.

The mean age (37.15 years) and male predominance (67.6%) in our cohort are consistent with the demographic profile reported in the literature. Wong et al. reported a mean age of 39 years, with a 78.6% male prevalence, while Müller et al. reported a mean ISS of 35 in their seminal series [2,13]. The high proportion of high-energy vehicular trauma, comprising pedestrian struck by vehicle (54.4%) and motor vehicle collision (27.9%), which together accounted for 82.3% of cases, is consistent with the established understanding that these injuries arise from high-velocity impact mechanisms [3,5,9].

The most powerful modifiable predictor of outcome in our cohort was the quality of acetabular reduction. A satisfactory Matta’s radiological score was associated with markedly lower odds of an unsatisfactory functional outcome (OR = 0.06; 95% CI: 0.01–0.56; *p* = 0.014), although the wide confidence interval and the exploratory nature of the analysis indicate that the precise magnitude of this protective effect should be interpreted with caution. This finding is consistent with, and extends, the established body of evidence on the importance of anatomical reduction in isolated acetabular fractures. Matta reported that anatomical reduction in the acetabulum was the most important factor determining long-term clinical outcomes after acetabular fracture surgery [14]. In the context of floating hip injuries specifically, Meena et al. found that clinical and radiological outcomes in type B floating hip injuries were significantly associated with the reduction quality on postoperative radiographs [11]. Our study corroborates these findings for type A injuries and quantifies the magnitude of the effect. The near-complete elimination of risk associated with anatomical reduction underscores the paramount importance of achieving precise articular restoration even in the setting of complex polytrauma.

Gänsslen et al. observed that transverse with posterior wall fractures of the acetabulum carry a significant risk of early joint failure even when anatomical congruency is achieved postoperatively, reporting a 32.8% joint failure rate in their series [16]. In our cohort, the transverse with posterior wall pattern was markedly overrepresented in the unsatisfactory outcome group. This concentration of complex fracture patterns in the poor outcome group likely reflects the inherent difficulty of achieving anatomical reduction in these fractures, as well as the cartilaginous damage sustained at the time of injury. Pavelka et al., in their series of 54 floating hip patients, similarly reported that imperfect reduction in the acetabulum, defined as residual displacement exceeding 2 mm, was the most common complication in acetabular fracture management and was strongly associated with the development of post-traumatic arthritis [19].

Sharma et al. reported that 80% of patients with both-column acetabular fractures managed with bi-columnar plating achieved excellent–good results, with a mean modified Harris hip score of 85.7. This further emphasizes that meticulous surgical technique and anatomical restoration are essential for favorable outcomes in complex acetabular injuries [20]. Our data suggest that this principle applies with even greater force in the floating hip scenario, where the biomechanical demands on the reconstructed acetabulum are compounded by the coexisting femoral fracture and its associated rehabilitation limitations.

Our finding that each one-unit increase in BMI was associated with a 1.5-fold increase in the odds of an unsatisfactory outcome is novel in the floating hip literature. The mean BMI in the unsatisfactory group was 29.09 ± 2.84 kg/m^2^, placing most patients in this group in the overweight-to-obese range as per the WHO criteria, compared with 23.95 ± 2.98 kg/m^2^ in the satisfactory group. Although the influence of obesity on outcomes following isolated acetabular or pelvic fractures has been examined in several studies, its role in the specific context of floating hip injuries has not been previously reported.

Several mechanistic pathways may explain the adverse influence of elevated BMI. Higher BMI increases the technical difficulty of surgical exposure and reduction, prolongs operative time, and increases blood loss, all of which may compromise the quality of acetabular reduction. Furthermore, obesity is associated with increased mechanical loading across the hip joint, potentially accelerating the development of post-traumatic arthritis in imperfectly reduced fractures. Additionally, obese patients often experience delayed mobilization and rehabilitation, which may contribute to joint stiffness, muscle atrophy, and inferior functional recovery. Wong et al. included BMI as a matching variable in their case–control analysis of floating hip injuries, recognizing its potential confounding influence, though they did not evaluate it as an independent prognostic factor [13]. Our results suggest that BMI should be considered in preoperative prognostic counseling and may warrant targeted perioperative optimization strategies in overweight and obese patients with floating hip injuries.

The presence of associated ipsilateral lower extremity fractures emerged as the strongest predictor of unsatisfactory outcome. This finding is striking and underscores the compounding effect of cumulative ipsilateral lower limb injuries on functional prognosis. In our cohort, 80% of patients in the unsatisfactory group had associated lower extremity fractures, compared with only 18.8% in the satisfactory group. These fractures included tibial, patellar, and foot injuries that impose additional constraints on weight bearing, rehabilitation and, ultimately, functional recovery.

This finding parallels the observations of Kokubo et al., who identified lower extremity fractures as a significant determinant of unsatisfactory short-term functional outcomes in patients with unstable pelvic ring fractures [12]. The magnitude of the effect in our cohort was substantially greater, which may reflect the synergistic impact of additional lower limb injuries superimposed upon an already biomechanically compromised extremity in the floating hip scenario. Zamora-Navas et al. described a floating hip as a “devastating injury” with a significant impact on patients’ quality of life that transcends the sum of individual injuries [7]. Our data quantify this observation: when the floating hip is further complicated by additional ipsilateral lower extremity fractures, the functional prognosis deteriorates dramatically.

Wong et al. demonstrated that floating hip cases were significantly more likely to have additional orthopedic injuries compared with matched controls, and that these additional injuries were associated with longer hospital stays and higher complication rates [13]. The clinical implication of our finding is that the identification of concomitant ipsilateral lower extremity fractures at the time of initial evaluation should prompt the treating team to anticipate a more guarded functional prognosis and to develop a comprehensive, multidisciplinary rehabilitation plan that addresses the entire kinetic chain.

Although nerve damage was significantly associated with unsatisfactory outcomes in the univariate analysis, it did not achieve independent significance in the multivariate model. This may be attributable to the confounding relationship between nerve damage and other injury severity markers, particularly the transverse with posterior wall fracture pattern and proximal femoral fractures, both of which are associated with posterior-type injury mechanisms that place the sciatic nerve at risk. Kokubo et al. reported that nerve damage was the most powerful predictor of both short-term (OR = 21.392) and long-term (OR = 66.926) functional outcomes in pelvic ring fractures [12]. The difference in the independent prognostic significance of nerve damage between our study and that of Kokubo et al. may reflect differences in patient populations and in the severity spectrum of neurological injuries encountered.

Proximal femoral fractures were predominant in the unsatisfactory group. Proximal femoral fractures with a floating hip injury may represent a higher-energy injury mechanism and may be associated with greater soft tissue disruption around the hip joint. Furthermore, proximal femoral fractures often require different rehabilitation protocols than shaft fractures, with more prolonged periods of restricted weight bearing that may impede functional recovery. Yang et al. noted that the injury mechanism in floating hip injuries typically involves a longitudinal force along the femoral shaft, causing an acetabular fracture first, followed by a femoral fracture, with residual force transmission determining the fracture level [9]. Suzuki et al. advocated for prioritizing femoral fixation in the surgical sequence when an acetabular fracture is present, recognizing that a stabilized femur facilitates both reduction maneuvers during acetabular surgery and early postoperative mobilization [4]. In our study, we initially fixed the femur, followed by the acetabulum.

Floating hip injuries result from high-energy trauma and are frequently accompanied by a wide spectrum of concomitant injuries involving multiple organ systems. In Matta’s landmark series, approximately 50% of patients with acetabular fractures sustained associated injuries, with extremity injuries being the most prevalent (35%), followed by head injuries (19%), chest injuries (18%), nerve palsy (13%), abdominal injuries (8%), genitourinary injuries (6%), and spinal injuries (4%) [14]. Similarly, in a large multicenter study involving 1273 patients with acetabular fractures, Kempegowda et al. identified head injuries in 20%, chest injuries in 20%, and abdominal and genitourinary injuries in 12% of patients who also had concomitant knee pathology [21]. In this study, thoracic injuries were observed in 36.8% of patients, followed by brain injuries (22.1%), abdominal injuries (23.5%), spinal injuries (16.2%), and genitourinary injuries (16.2%). None of these associated organ injuries demonstrated a statistically significant difference between the satisfactory and unsatisfactory outcome groups, suggesting that while systemic injuries contribute to overall morbidity, they may not be the primary determinants of radiographic and functional outcome following surgical fixation of floating hip injuries. This finding is consistent with the existing literature, which emphasizes that the quality of articular reduction and fracture pattern are more reliable predictors of long-term outcomes than the presence of concomitant systemic injuries [22].

Knee ligament injuries constitute an often overlooked but clinically significant entity in patients with ipsilateral femur fractures and acetabular fractures. In a multicenter study of 1273 acetabular fractures, Kempegowda et al. found that 15% of patients developed ipsilateral knee symptoms within one year, with 25% of these attributed to ligamentous lesions, and posterior cruciate ligament (PCL) injuries being the most frequent pattern [21]. Odagiri et al. recently demonstrated that hip dislocation–fracture significantly increases the risk of ipsilateral knee injury, particularly PCL injury, and advocated for systematic knee evaluation, including MRI, in all patients with acetabular fracture–dislocations [23]. Regarding femoral shaft fractures specifically, the reported incidence of concomitant ipsilateral knee ligament injury varies considerably, from 5.3% to 48%, depending on the diagnostic methods employed and the timing of evaluation [24,25]. In this study, ipsilateral knee ligament injury was observed in 20.6% of patients (18.8% in the satisfactory group and 25% in the unsatisfactory group), while contralateral knee ligament injury was relatively rare, at 4.4% (4.2% vs. 5%, respectively). Although neither ipsilateral nor contralateral knee ligament injuries reached statistical significance in relation to outcome, their presence warrants careful clinical attention, as delayed recognition of ligamentous pathology can lead to secondary meniscal and chondral damage, chronic instability, and impaired rehabilitation [21,24]. We advocate for a thorough clinical and, when feasible, MRI-based evaluation of both knees in all patients presenting with floating hip injuries, particularly in the context of high-energy mechanisms such as motor vehicle collisions and dashboard-type injuries.

The overall satisfactory outcome rate of 70.6% in our cohort is consistent with published data on floating hip injuries. Pavelka et al. reported excellent results in 80% of type A floating hip injuries and a combined excellent–very good rate of 79% for type B injuries [19]. Meena et al. achieved excellent or good clinical and radiological outcomes in more than 60% of type B cases [11]. Burd et al. reported satisfactory outcomes in most floating hip patients but noted that outcomes were influenced by the specific combination of pelvic or acetabular and femoral fracture types [6]. Our complication rate of 26.5% is lower than the 53.8% reported by Wong et al. for surgically managed floating hip cases, a difference that may reflect variations in case population, surgical technique, and definitions of complications across studies [13].

This study’s findings carry several important clinical implications. First, the overwhelming importance of achieving anatomical acetabular reduction reinforces the need for experienced pelvic and acetabular surgeons to manage these complex injuries, ideally at specialized tertiary trauma centers. Patients presenting with Müller type A floating hip injuries should be transferred to high-volume centers with dedicated pelvic and acetabular surgery expertise, as the quality of articular reduction, identified as the most powerful modifiable predictor of outcome, directly depends on surgeon experience and institutional infrastructure. Second, the identification of BMI as an independent prognostic factor suggests a potential role for perioperative metabolic optimization and aggressive postoperative rehabilitation in overweight and obese patients. Practically, this may include preoperative nutritional assessment and optimization, structured weight management counseling initiated during the rehabilitation phase, and a more intensive, closely supervised physiotherapy program tailored to the biomechanical demands imposed by elevated body weight on the reconstructed hip joint. Anesthesiological and surgical teams should also anticipate greater technical difficulty in achieving anatomical reduction in obese patients, with corresponding implications for operative planning, patient positioning, and implant selection. Third, the recognition that associated lower extremity fractures dramatically worsen the prognosis should inform prognostic discussions with patients and their families and should guide the development of comprehensive, multidisciplinary treatment and rehabilitation plans. At the time of initial evaluation, the identification of concomitant ipsilateral lower extremity fractures should prompt the treating team to explicitly communicate a guarded long-term functional prognosis and to develop a rehabilitation strategy that addresses the entire ipsilateral kinetic chain, from the reconstructed acetabulum through the femur to the distal extremity injuries, in a coordinated and sequential manner. Early involvement of a multidisciplinary rehabilitation team, including physiotherapy, occupational therapy, and pain management specialists, is advisable in this subgroup. Fourth, the combination of these three independent factors may enable the construction of a clinical prediction tool to stratify patients at the time of initial presentation, thereby guiding treatment intensity and resource allocation. Future prospective validation of a scoring system incorporating BMI, the presence of associated ipsilateral lower extremity fractures, and anticipated Matta reduction quality could provide clinicians with an objective, readily applicable instrument for outcome prediction at the point of care.

Several aspects of this study contribute to the existing literature in a novel manner. To our knowledge, this is the first study to demonstrate that BMI is an independent prognostic determinant of long-term functional outcomes specifically in Müller type A floating hip injuries, and to quantify this effect with a multivariate odds ratio. Prior analyses either did not include BMI as a candidate variable, or treated it solely as a matching covariate [14]. Furthermore, while the prognostic importance of acetabular reduction quality has been established in the literature on isolated acetabular fractures, this study is among the first to quantify this association—and, specifically, the magnitude of the protective effect of anatomical reduction within the context of a floating hip injury. Finally, the identification of associated ipsilateral lower extremity fractures as the strongest independent predictor in our cohort, with a substantially elevated but imprecisely estimated odds ratio (OR = 29.02; 95% CI: 2.83–297.67), has not been previously reported in this specific injury subtype. The wide confidence interval reflects the limited events-per-variable ratio and indicates that, although the direction and clinical significance of this association appear robust, the precise magnitude of the effect should be regarded as exploratory and requires confirmation in larger, multicenter cohorts. These findings provide a preliminary prognostic framework that extends previously established principles and may inform surgical counseling and rehabilitation planning.

This study has several limitations that warrant acknowledgment. First, its retrospective design introduces the possibility of selection bias and information bias inherent to chart review. Second, the sample size of 68 patients, while reasonable for this rare injury, limits the statistical power of the multivariate analysis and may explain the wide confidence intervals observed for some predictors. In particular, the events-per-variable ratio of approximately 6.7 (20 outcome events divided by 3 independent predictors in the final model) falls below the traditionally recommended threshold of 10:1, which increases the risk of model overfitting and likely contributes to the exceptionally wide confidence interval observed for the associated lower extremity fracture predictor (OR = 29.02; 95% CI: 2.83–297.67). Therefore, these estimates should be interpreted with appropriate caution, and the precision of effect size estimates should be improved in future studies with larger sample sizes. Third, this study was conducted at a single institution, which may limit the generalizability of the findings to other settings with different patient populations, surgical expertise, or perioperative protocols. Fourth, the dichotomization of the Majeed score for the group comparison, while clinically meaningful, may result in some loss of discriminatory information. Fifth, this study spans a 20-year treatment period (2000–2019), during which incremental changes in surgical implants, perioperative care protocols, and rehabilitation strategies may have occurred. Although standardized operative principles and consistent outcome assessment methods were maintained throughout the study period at a single institution, we cannot entirely exclude the potential influence of temporal variation on outcomes. Future prospective multicenter studies with larger cohorts and comprehensive patient-reported outcome measurement assessment are warranted to validate and extend these findings.

## 5. Conclusions

In patients with Müller type A floating hip injuries, elevated body mass index, the presence of associated ipsilateral lower extremity fractures, and poor quality of acetabular reduction, as assessed via Matta’s radiological scoring system, are independent determinants of unsatisfactory long-term functional outcomes. The quality of acetabular reduction emerged as the most powerful modifiable protective factor, with anatomical reduction associated with markedly lower odds of an unsatisfactory outcome, while the presence of additional ipsilateral lower limb injuries appears to compound functional prognosis. Given the exploratory nature of the multivariate estimates and the limited events-per-variable ratio, these findings should be regarded as hypothesis-generating and require external validation in larger, multicenter cohorts. They may nonetheless inform surgical decision-making, patient counseling, and rehabilitation planning in this challenging injury pattern.

## Figures and Tables

**Table 1:** Revised trauma score variables.

Coded Numerical Value	Systolic Blood Pressure	Respiratory Rate	Glasgow Coma Scale
4	>89	10–29	13–15
3	76–89	>29	9–12
2	50–75	6–9	6–8
1	1–49	1–5	4–5
0	0	0	3

During triage, patients with an RTS of 11–12 are regarded as urgent, patients with an RTS of 3–10 are considered immediate, and a score of 3 or less is often indicative of mortality.

**Table 2:** Classification of acetabulum fractures based on the Judet–Letournel classification system and corresponding surgical procedures.

Fracture Type	Count (%)	Surgical Approach
Anterior wall	6 (8.8%)	Modified Stoppa
Anterior column	10 (14.7%)	Modified Stoppa (6); ilioinguinal (4)
Both column	9 (13.2%)	Ilioinguinal (1); both (5); modified Stoppa + lateral window (3)
Posterior wall	9 (13.2%)	KL
Posterior column	6 (8.8%)	KL
T-type	3 (4.4%)	KL (1); both (2)
Transverse	6 (8.8%)	Ilioinguinal (1); both (5)
Transverse + posterior wall	19 (27.9%)	Ilioinguinal + KL (4); both (15)

KL: Kocher–Langenbeck; both: modified Stoppa + Kocher–Langenbeck.

**Table 3:** Baseline characteristics and between-group comparisons of categorical variables in patients with satisfactory versus unsatisfactory long-term functional outcomes.

Variables n (%)	Satisfactory (n = 48)	Unsatisfactory (n = 20)	*p*
Sex			0.403
Female	17 (35.4)	5 (25)	
Male	31 (64.6)	15 (75)	
Side			0.135
Left	17 (35.4)	11 (55)	
Right	31 (64.6)	9 (45)	
Acetabular fracture type			0.001 *
Anterior wall	6 (12.5)	0 (0)	
Anterior column	8 (16.7)	2 (10)	
Both column	6 (12.5)	3 (15)	
Posterior wall	9 (18.8)	0 (0)	
Posterior column	4 (8.3)	2 (10)	
T-type	3 (6.3)	0 (0)	
Transverse	6 (12.5)	0 (0)	
Transverse + posterior wall	6 (12.5)	13 (65)	
Femur fracture level			0.001 *
Distal	9 (18.8)	1 (5)	
Proximal	11 (22.9)	15 (75)	
Shaft	28 (58.3)	4 (20)	
Associated lower extremity fracture			0.001 *
Yes	9 (18.8)	16 (80)	
None	39 (81.3)	4 (20)	
Associated upper extremity fracture			0.919
Yes	15 (31.3)	6 (30)	
None	33 (68.8)	14 (70)	
Nerve injury			0.001 *
Yes	4 (8.3)	11 (55)	
None	44 (91.7)	9 (45)	
Vascular injury			0.879
Yes	2 (4.2)	1 (5)	
None	46 (95.8)	19 (95)	
Associated injuries			
Brain			0.706
Yes	10 (20.8)	5 (25)	
None	38 (79.2)	15 (75)	
Spine			0.865
Yes	8 (16.7)	3 (15)	
None	40 (83.3)	17 (85)	
Thorax			0.721
Yes	17 (35.4)	8 (40)	
None	31 (64.6)	12 (60)	
Abdomen			0.417
Yes	10 (20.8)	6 (30)	
None	38 (79.2)	14 (70)	
Genitourinary			0.372
Yes	9 (18.8)	2 (10)	
None	39 (81.3)	18 (90)	
Fracture mechanism			0.031 *
Motor vehicle collision	9 (18.8)	10 (50)	
Pedestrian struck by vehicle	29 (60.4)	8 (40)	
Fall from height	10 (20.8)	2 (10)	
Ligament injury in the ipsilateral knee			0.561
Yes	9 (18.8)	5 (25)	
None	39 (81.3)	15 (75)	
Ligament injury in the contralateral knee			0.879
Yes	2 (4.2)	1 (5)	
None	46 (95.8)	19 (95)	
Smoking habit			0.859
Smoker	13 (27.1)	5 (25)	
Non-smoker	35 (72.9)	15 (75)	

* Significant at 0.05 level; chi-square test and Bonferroni correction for multiple comparisons.

**Table 4:** Surgical algorithm and fixation methods applied according to femoral fracture level.

Fracture Region (n)	Fracture Type (n)	Fixation Method	n
Proximal (26)	Femoral neck (2)	7.3 mm cannulated screws	1
		DHS	1
	Intertrochanteric (16)	DHS	3
		PFN	10
		Proximal femur locking plate	3
	Subtrochanteric (8)	PFN	3
		AIN	5
Shaft (32)	—	AIN	24
		RIN	8
Distal (10)	Extra-articular (6)	RIN	3
		Distal femur lateral anatomic plate	3
	Intra-articular (4)	Distal femur lateral anatomic plate	4

n: the number of fractures; DHS: dynamic hip screw; PFN: proximal femoral nail.

**Table 5:** Baseline demographic, clinical, and outcome variables: between-group comparison of continuous variables in patients with satisfactory versus unsatisfactory long-term functional outcomes.

Variables	Satisfactory (n = 48)	Unsatisfactory (n = 20)	*p*
	Mean ±SD	Mean ±SD	
Age	36.67 ± 11.69	38.3 ± 13.19	0.762
Revised trauma score	6.92 ± 0.84	6.75 ± 0.84	0.506
Age-adjusted Charlson comorbidity index	2.06 ± 1.48	2.1 ± 1.74	0.506
Body mass index	23.95 ± 2.98	29.09 ± 2.84	0.001 *
Time to definitive surgery (day)	4.4 ± 2.18	4.1 ± 2.22	0.460
Length of hospital stay	16.65 ± 13.29	17.45 ± 10.92	0.568
Majeed score	79.63 ± 6.54	60.1 ± 3.89	0.001 *

* Significant at 0.05 level; Mann–Whitney U test. The Majeed score is presented for descriptive completeness, as it defines the binary outcome variable used for group allocation; it was excluded from the multivariate regression model on conceptual grounds.

**Table 6:** Assessment of prerequisites for regression analysis.

Coefficients ^a^			
Model		Collinearity Statistics	
		Tolerance	VIF
1	Fracture level	0.897	1.115
	Associated lower extremity fracture	0.692	1.445
	Nerve damage	0.698	1.433
	Fracture mechanism	0.883	1.133
	Matta’s radiological scoring system	0.515	1.942
	Majeed score	0.386	2.592
	Body mass index	0.553	1.808

^a^ Dependent variable: outcome group.

**Table 7:** Multivariate binary logistic regression analysis of independent predictors of unsatisfactory functional outcome.

Variable	OR [95% CI]	*p*-Value
Matta’s radiological score	0.06 [0.01–0.56]	0.014 *
Body mass index	1.50 [1.05–2.15]	0.025 *
Associated lower extremity fracture	29.02 [2.83–297.67]	0.005 *

OR = odds ratio; CI = confidence interval. * Statistically significant at *p* < 0.05.

## Data Availability

Data are available upon request due to restrictions (e.g., privacy, legal, or ethical reasons).

## Abbreviations

The following abbreviations were used in this manuscript:

ISS	Injury severity score
ACCI	Age-adjusted Charlson comorbidity index
RTS	Revised trauma score
AVN	Avascular necrosis
HO	Heterotopic ossification
THA	Total hip arthroplasty
TKA	Total knee arthroplasty
